# ICU vampires and anemia

**DOI:** 10.1186/s13054-015-0748-5

**Published:** 2015-01-28

**Authors:** Tamra Ranasinghe, William D Freeman

**Affiliations:** Department of Neuroscience, Mayo Clinic, 4500 San Pablo Rd, Jacksonville, FL 32224 USA; Department of Neurology, Mayo Clinic, 4500 San Pablo Rd, Jacksonville, FL 32224 USA; Department of Neurosurgery, Mayo Clinic, 4500 San Pablo Rd, Jacksonville, FL 32224 USA; Department of Critical Care, Mayo Clinic, 4500 San Pablo Rd, Jacksonville, FL 32224 USA

We read with great interest the article by Fischer and colleagues in a recent issue of *Critical Care* [1]. The authors make compelling arguments about the different types of blood loss in inpatients and outpatients and describe potential solutions. We agree with the authors about this ‘vampirism’ , which is of major importance in patient care. We add that another issue leading to excessive blood loss in hospitals is computerized order entry, which at our center can cause daily complete blood cell counts, electrolytes, and other labs to be drawn by phlebotomists. We feel it is important to teach trainees (medical students, residents and attendings) that it may be perfectly acceptable to not have morning labs on morning rounds with the attending. Only labs that are clinically relevant or guide management should be ordered. Labs just for the sake of having labs for morning rounds should not occur without good clinical reason. However, in today’s cost-conscious health-care culture [2], we must make an active effort. Finally, we find that pediatric vial-sized blood draws may be another potential way to lessen this blood loss [3]. We appreciate the authors’ other solutions for this ‘vampiric anemia’ and we hope they will be adopted by others to improve patient care.
